# Leveraging long acting reversible contraceptives to achieve FP2020 commitments in sub-Saharan Africa: The potential of implants

**DOI:** 10.1371/journal.pone.0195228

**Published:** 2018-04-09

**Authors:** Katherine Thanel, Danielle Garfinkel, Christina Riley, Keith Esch, Woldemariam Girma, Tadele Kebede, Gaby Kasongo, Kayode Afolabi, Amanda Kalamar, Sarah Thurston, Kim Longfield, Jane Bertrand, Bryan Shaw, on behalf of the FPwatch Group

**Affiliations:** 1 Population Services International, Washington, D.C., United States of America; 2 Population Services International – Ethiopia, Addis Ababa, Ethiopia; 3 Federal Ministry of Health, Addis Ababa, Ethiopia; 4 Association de Santé Familiale, Kinshasa, Democratic Republic of Congo; 5 Federal Ministry of Health, Ibadan, Nigeria; 6 Independent Consultant, Washington, D.C., United States of America; 7 Department of Global Health Systems and Development, Tulane University School of Public Health and Tropical Medicine, New Orleans, Los Angeles, United States of America; 8 Institute for Reproductive Health, Georgetown University, Washington, D.C., United States of America; Population Council, KENYA

## Abstract

**Background:**

In developing regions, an estimated 214 million women have an unmet need for family planning. Reaching Family Planning 2020 (FP2020) commitments will require a shift in modern contraceptive promotion, including improved access to long-acting reversible contraceptives (LARCs). Until now, a lack of market data limited understanding of the potential of LARCs to increase contraceptive access and choice.

**Methods:**

From 2015, the FPwatch Project conducted representative surveys in Ethiopia, Nigeria, and Democratic Republic of Congo (DRC) using a full census approach in selected administrative areas. In these areas, every public and private sector outlet with the potential to sell or distribute modern contraceptives was approached. In outlets with modern contraceptives, product audits and provider interviews assessed contraceptive market composition, market share, availability, price, and outlet readiness to perform services.

**Results:**

Fifty-four percent of outlets in Ethiopia had LARC commodities or services available at the time of the survey, versus 7% and 8% of outlets in Nigeria and DRC, respectively. When present, LARCs were usually available with at least two other methods (99%, 39%, and 84% of public health facilities in Ethiopia, Nigeria and DRC, respectively). Many public facilities had both implants and IUDs in stock (76%, 47%, and 53%, respectively). Lack of readiness to provide LARCs was mostly due to a lack of equipment, private room, or the commodity itself. Market share for implants in the public sector was 60%, 53%, and 37% of Couple Years of Protection (CYP) in Ethiopia, Nigeria, and DRC.

**Discussion:**

Limited availability of LARCs in Nigeria and DRC restricts contraceptive choice and makes it difficult for women to adopt and use modern contraception consistently. Brand-specific subsidies, task shifting, and promotion of methods that require less equipment and training are promising strategies for increasing uptake. Substantial government investment is required to improve availability and affordability. Investment in implants should be prioritized to make progress towards FP2020 commitments.

## Introduction

In developing regions, an estimated 214 million women have an unmet need for family planning, [1] leading to excessive health costs to individuals and families as well as socioeconomic consequences for nations. [2] To reduce unmet need and prevent unintended pregnancies, the Family Planning 2020 (FP2020) Initiative was launched in 2012. FP2020 is a global partnership of family planning stakeholders which aims to add 120 million additional users of modern contraceptives by 2020 in the world’s least-developed countries by offering choice among a range of safe and effective methods. [3] Reaching FP2020 commitments will require a strategic shift in modern contraceptive promotion, including improved access to long-acting reversible contraceptives (LARCs). LARCs are highly effective in preventing pregnancy, less likely to be discontinued compared with short acting methods, and offer the benefits of convenience and comparatively low cost over time. [4] Interest in improving access and use of LARCs was featured prominently at the Fourth International Conference on Family Planning and is increasingly a priority in sub-Saharan Africa (SSA). [5]

Globally, SSA has the highest proportion of unmet need, with more than 1 in 5 women in need of modern contraceptives. [1] Contraceptive supply and demand are dependent on each other; a limited method mix can contribute to variable demand for modern contraceptives. [6] Despite high levels of effectiveness and acceptability of contraceptive implants and intrauterine devices (IUDs), [7] LARCs still account for only a small percentage of the method mix in many SSA countries (see S1 Table).

Achieving global FP2020 commitments will depend on the successes of countries with large populations and high unmet need. In SSA, high-priority countries include Ethiopia, Nigeria, and the Democratic Republic of Congo (DRC). These countries are characterized by different contraceptive markets and method mixes, as reported in the PMA2020 semi-annual briefs. [8] While Ethiopia has made rapid advances in increasing modern contraceptive rates (mCPR) with implants and injectables, [8] progress toward national FP2020 commitments (see S1 Table) has been slower in Nigeria and DRC where users are more heavily dependent on shorter acting methods. [8, 9]

Several initiatives are underway for improving access to and use of LARCs, with Ethiopia having the most comprehensive strategy of the three countries. There is public sector facility strengthening and community-based provision of Implanon NXT^®^ in Ethiopia [10] and Nigeria, [11] and private sector social franchising and mobile outreach service delivery in Ethiopia, [11] Nigeria, [11] and DRC. [12] In 2015 and 2016, pilot programs were launched for task shifting [13], implants and IUDs to Community Health Extension Workers (CHEWs) in Nigeria [14] and implants to medical and nursing students in DRC. [15] In Ethiopia, substantial financial commitments at the national and regional levels have bolstered programs and commodity procurement across the country. [16] Likewise, institutionalized task shifting for implant distribution to Health Extension Workers (HEWs) has made LARCs available throughout the country (see S2 Table). These additional efforts in Ethiopia have expedited progress against FP2020 commitments and distinguished Ethiopia as a family planning success story. [17]

There has been a gap in systematically collected data on contraceptive markets and LARCs, which hinders policymakers’ ability to improve method diversity and choice. In recent years PMA2020 has shed light on method mix, contraceptive prevalence and other market indicators that help fill this void and reinforce the findings of FPwatch. [8] In this paper, we examine market composition and volume alongside LARC availability, service readiness, and price to understand where women are accessing LARCs and how LARCs can be leveraged to help countries achieve their FP2020 commitments.

## Methodology

The FPwatch Project provided insight into contraceptive markets and estimates for key family planning indicators in Ethiopia, Nigeria, and DRC. FPwatch surveys generated nationally- and regionally-representative data from a cross-section of outlets to complement findings from other health facility surveys, like the Performance, Monitoring and Accountability 2020 (PMA2020) surveys. [18] In addition, rigorous analysis on large sample sizes increased confidence in estimates while a systematic sampling approach provided a snapshot of the total contraceptive market.

### Study design and sample selection

Data were collected in the second half of 2015. In Ethiopia, the geopolitical areas included the four most populous regions where 85% of the population resides: Amhara; Oromia; Southern Nations, Nationalities, Peoples’ Region (SNNP); and Addis Ababa. The Nigerian survey was nationally representative: outlets from all six geopolitical zones were included. Two provinces were intentionally sampled in DRC to ensure the inclusion of one primarily urban and one primarily rural province, Kinshasa and Katanga, respectively. In 2015, DRC’s 11 original provinces were divided into 26; FPwatch used the pre-2015 boundaries in province selection.

Sample size requirements were based on estimates for the proportion of outlets with three or more modern methods of contraception in stock on the day of the survey at 95% confidence. For the purposes of this survey, our definition of modern contraceptives included oral contraceptives, emergency contraceptives, injectable contraceptives, contraceptive implants, and IUDs. This indicator was selected for its high relevance to FP2020 commitments (see S1 Table). One- or two-stage sampling was conducted using probability proportional to size, with representative clusters of approximately 10,000 to 15,000 people. [19] In DRC, a booster sample was included for public health facilities and pharmacies to adjust for insufficient numbers of these outlet types. The detailed sampling strategy for each country is included in S3 and S4 Tables.

Data collection lasted six to eight weeks and field teams used a full census approach to identify potential outlets: data collectors met with local authorities to produce sketch maps of the areas’ potentially eligible outlets or walked down all streets and paths of selected administrative units to find them.

Every public and private sector outlet with the potential to sell or distribute modern methods was screened for eligibility. Public sector outlets included hospitals, health centers, community health workers, and, in the case of DRC, representative private clinics in areas with no public facility. The private sector included private clinics, pharmacies, drug stores, and general retailers, except in DRC where general retailers were not surveyed. General retailers were not screened in DRC because these outlets do not stock contraceptives beyond condoms. Bars, brothels, and police and military hospitals that did not serve the general public were excluded from the study. A detailed description of outlet types and their capabilities for LARC provision is provided in S2 Table.

Outlets must have met one of the following criteria to be eligible for a full interview and product audit: 1) stocked at least one modern contraceptive method other than condoms (oral contraceptives, emergency contraceptives, injectable contraceptives, contraceptive implants, or IUDs) on the day of the survey; 2) had one or more types of modern contraceptives other than condoms available within the three months preceding the survey; or 3) offered provider-dependent contraceptive services, including contraceptive injections, implant insertions, IUD insertions, and/or male/female sterilization.

### Data collection

At all eligible outlets, providers were invited to join the study after giving verbal informed consent. Data collectors used paper questionnaires to complete audits of relevant product information, including product brand name, generic name, active ingredient and corresponding strength, manufacturer name, and country of manufacture. For each brand, providers/outlet staff reported on volume distributed during the previous month, stock out during the previous three months, and retail and wholesale price. At outlets stocking or providing services for provider-dependent methods (these include contraceptive injections, implants, IUDs, and male and female sterilization), another set of questions assessed provider readiness to perform the service, including implant and IUD insertions and removals. Questionnaires were translated into local languages, reviewed by field teams and back-translated.

### Data analysis

Data were double entered into Microsoft Access (Microsoft Corporation, Redmond, Washington, US). All data analyses were conducted in Stata 14 and weighted using the inverse cluster selection probability (StataCorp, College Station, Texas, USA).

A set of standard indicators was calculated for modern contraceptive methods, namely market composition, availability, stock-out, and median price. Total modern contraceptive market share is the relative proportion of modern contraceptives sold or distributed (i.e. volume) in the past month and was calculated using couple years of protection (CYP), [20] the estimated protection a contraceptive provides over a one-year period. This approach standardized impact across products that differ in the amount/length of protection against pregnancy. Information on consumer price was collected in local currency. Price was then converted into USD using the average exchange rate during data collection and reported as cost per CYP.

Service readiness for implant and IUD insertion was measured by the availability of: 1) the commodity; 2) the appropriate provider credentials according to national policy; and 3) the necessary equipment for insertions. [20] For removal service readiness, only provider credentials and removal equipment were measured. Although components to measure service readiness for removals were present, there was no specific question on the questionnaire related to provision of removal services or volume of removal services. Reasons for lack of readiness, such as lack of the commodity or equipment, were assessed only among outlets offering the service.

This study was approved by the National Research Ethics Review Committee at the Ministry of Science and Technology in Ethiopia, the National Health Research Ethics Committee of Nigeria at the Federal Ministry of Health, and the Comité Ethique, Ecole de Santé Publique, Université de Kinshasa in DRC.

#### Data sharing

All study data are available on the Harvard Dataverse as follows:

DRC URL: http://dx.doi.org/10.7910/DVN/OJD10N

DRC DOI: doi:10.7910/DVN/OJD10N

Ethiopia URL: http://dx.doi.org/10.7910/DVN/JRTCW5

Ethiopia DOI: doi:10.7910/DVN/JRTCW5

Nigeria URL: http://dx.doi.org/10.7910/DVN/2HRQON

Nigeria DOI: doi:10.7910/DVN/2HRQON

#### Role of the funding source

The funders of the study had no role in study design, data collection, data analysis, data interpretation, or writing of the report. The corresponding author had full access to all the data in the study and had final responsibility for the decision to submit for publication.

## Results

### LARC commodity and service availability and method diversity

**Fig 1:**
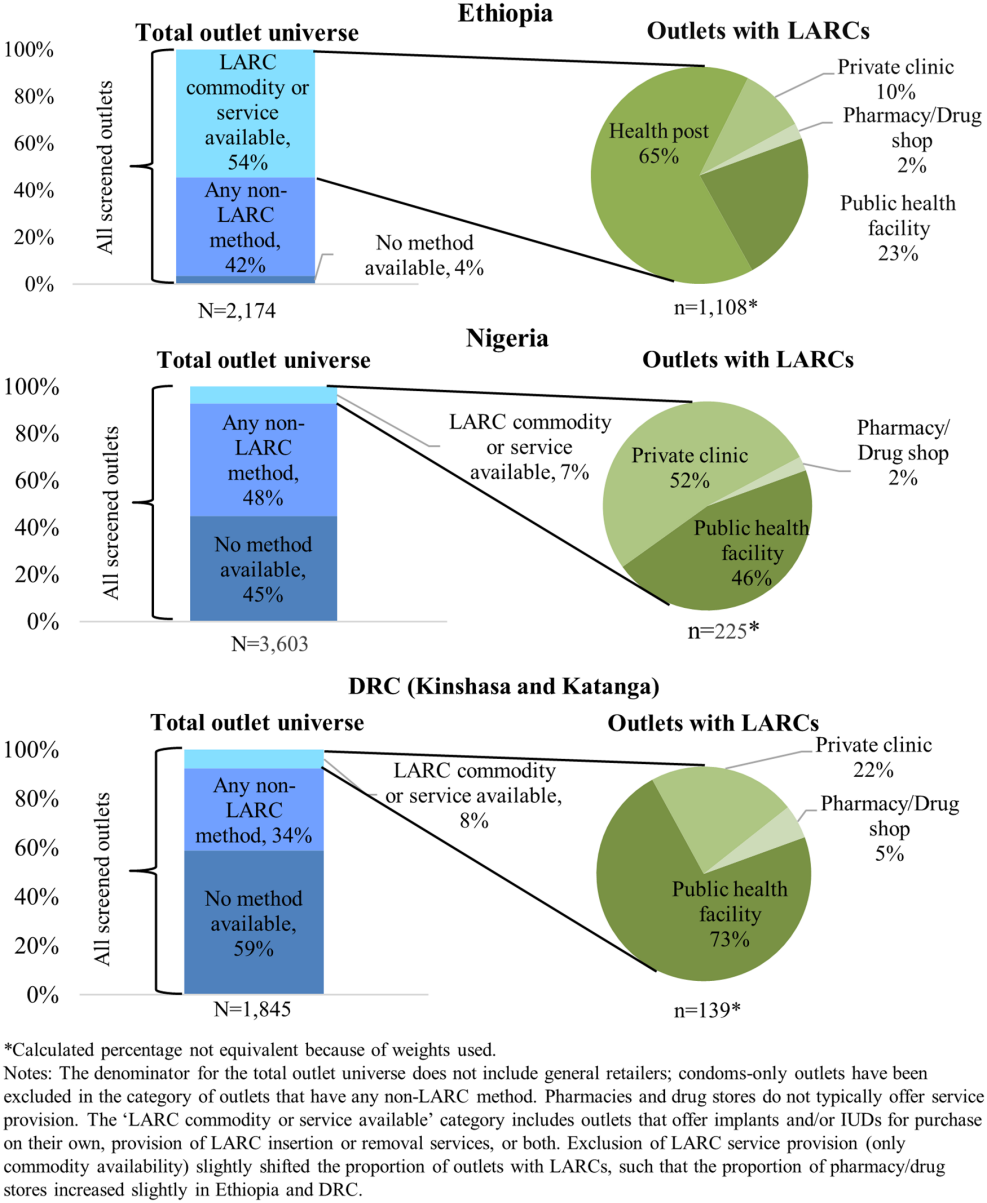
LARC commodity or service availability by outlet type and country.

Just over half of all outlets in Ethiopia had a LARC commodity or service available at the time of the survey. This did not include general retailers, which almost never carried contraceptives other than condoms. Sixty-five percent of LARC-stocking outlets were health posts staffed by HEWs and another 23% were public health facilities for a total of 88% of LARC-providing outlets in the public sector (Fig 1). In Nigeria and DRC, only 7% and 8% of outlets respectively had a LARC commodity or service available at the time of the survey. Nearly half of Nigeria’s LARC-stocking outlets and three quarters of DRCs were public facilities.

**Fig 2:**
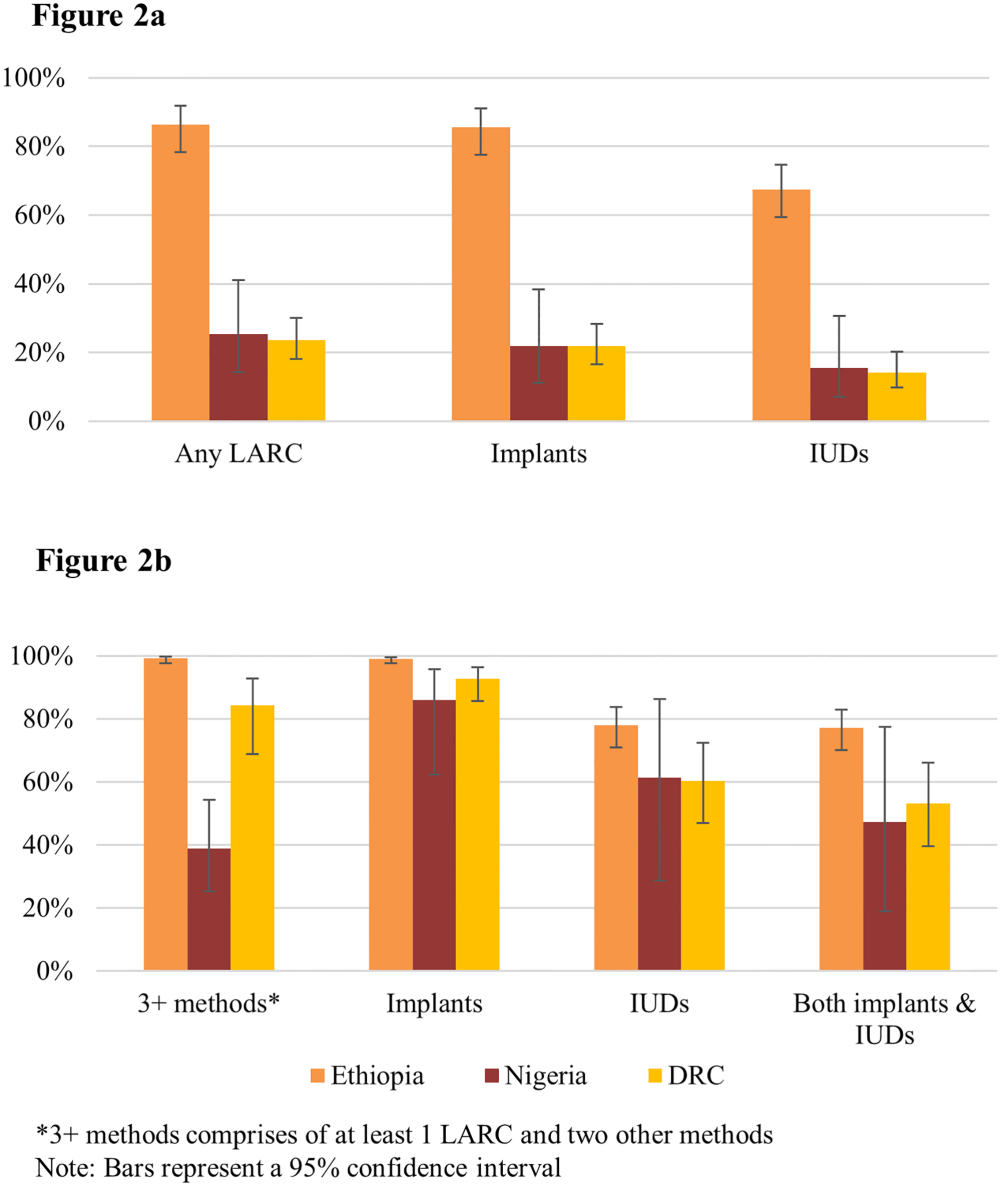
a: Method availability, among all public health facilities. b. Method diversity, among public health facilities with at least 1 LARC.

Highly variable availability of LARCs was observed within public health facilities across the three countries. Ethiopian public health facilities were more likely to have LARC methods in stock than those in Nigeria and DRC. Implants were stocked by 86% of public health facilities in Ethiopia compared with 22% in both Nigeria and DRC. IUDs were stocked in 68% of Ethiopian public health facilities but only in 16% and 14% of these facilities in Nigeria and DRC (Fig 2a). In Nigeria, the only country with higher LARC availability in private clinics than in public facilities, implants and IUDs were stocked in 24% and 20% of private clinics, respectively. [19]

Despite very low levels of LARC availability in facilities in Nigeria and DRC, when present, LARCs were usually available within a range of methods in the three countries: 99% of public health facilities with LARCs in Ethiopia, 39% in Nigeria, and 84% in DRC had at least two other methods available (Fig 2b). Over 77% of LARC-stocking facilities in Ethiopia and approximately half in Nigeria (47%) and DRC (53%) had both implants and IUDs in stock. Two-thirds of implant-stocking outlets in Ethiopia and Nigeria had more than one brand of implant available, whereas in DRC there was rarely a choice among implants.

### Readiness of outlets providing LARC services

Among public health facilities with implant insertion services available, service readiness was 77% in Ethiopia, 73% in Nigeria, and 44% in DRC (Table 1). In Ethiopia and DRC, most facilities that were not service ready lacked equipment, did not have a private room, or were missing consumables such as iodine. In Nigeria, lack of implant commodities was the most common impediment to service readiness (54%). Most facilities that were service ready for implant insertions were also equipped for removals.

**Table 1:** Service readiness to provide provider-dependent contraceptive insertion services and reasons for lack of service readiness, among outlets reportedly offering the procedure, by outlet type and country.

		Ethiopia	Nigeria	DRC
		Public Health Facility	Public Health Facility	Public Health Facility
Proportion of outlets offering service, with^†^:		% [95% CI]	% [95% CI]	% [95% CI]
***Contraceptive implant insertion service***				
Among those reportedly offering the service:		N = 253	N = 70	N = 221
	Not service ready	22·6	27·0	55·9
		(16·4–30·4)	(10·8–53·0)	(43·6–67·5)
Among those not service ready		n = 47	n = 34	n = 105
	Lacking commodity	13·5	53·6	59·3
		(5·6–29·0)	(25·6–79·5)	(41·9–74·5)
	Lacking credentials	3·2	37·2	9·1
		(0·8–11·9)	(14·4–67·7)	(2·8–25·7)
	Lacking equipment	88·6	18·8	64·5
		(72·6–95·8)	(8·4–37·0)	(49·0–77·5)
Of those lacking the equipment		n = 38	n = 17	n = 59
	Lacking private room	70·8	17·3	50·9
		(52·3–84·3)	(4·1–50·5)	(30·7–70·8)
	Lacking trocar	18·5	40·8	62·9
		(7·7–38·0)	(18·8–67·2)	(45·7–77·4)
	Lacking iodine	23	85·2	19·4
		(12·0–39·6)	(59·0–95·8)	(8·7–38·1)
***IUD insertion service***				
Among those reportedly offering the service:		n = 195	n = 72	n = 149
	Not service ready	22·4	53·4	73·8
		(14·7–32·8)	(17·5–86·1)	(58·7–84·7)
Among those not service ready		n = 37	-	n = 93
	Lacking commodity	19·3	83·4	46·3
		(9·7–34·8)	(52·9–95·7)	(27·0–66·8)
	Lacking credentials	1·6	10·4	9·3
		(0·2–10·6)	(2·2–37·4)	(2·3–31·2)
	Lacking equipment	82·5	24·8	72·6
		(67·1–91·6)	(6·1–62·4)	(55·4–85)
Of those lacking the equipment		n = 28	-	n = 57
	Lacking private room	57·1	31·6	42
		(33·8–77·6)	(6·4–75·8)	(15·8–73·6)
	Lacking exam table	3·5	4·8	18·9
		(0·8–13·9)	(1·2–17·9)	(8·3–37·3)
	Lacking iodine	15·5	50·2	4·6
		(5·0–39·2)	(18·0–82·2)	(1·0–19·5)
	Lacking tenaculum	24·7	58·5	35·0
		(10·2–48·7)	(24·0–86·3)	(19·0–55·2)
	Lacking speculum	1·7	13·9	9·3
		(0·2–11·1)	(2·9–46·8)	(2·5–29·1)
	Lacking uterine sound	26·5	75·7	39·6
		(11·5–49·9)	(48·5–91·1)	(18·2–66·0)

^†^ Full service readiness is defined as having available: 1. The commodity; 2. A provider with credentials meeting the guidelines to perform the service; and 3. A minimum set of sentinel equipment (http://www.cpc.unc.edu/measure/prh/rh_indicators/specific/long-acting-permanent-methods/percent-of-facilities-with-appropriate) for providing the service.

Note: Service readiness data for removals was generally similar to data for insertions. Implant removals require forceps and a scalpel, and IUD removals require a string retriever in addition to the listed equipment for insertion. There was no commodity requirement for removal.

Among public health facilities offering IUD insertions, service readiness was highest in Ethiopia (78%) versus 47% in Nigeria and 26% in DRC. Among facilities not service-ready, the most common obstacle was a lack of IUD commodities: 19% of facilities in Ethiopia, 83% in Nigeria, and 46% in DRC. In DRC and Nigeria, facilities were more likely to be service ready for IUD removals than for insertions, which require presence of the contraceptive commodity. Among facilities not service ready for removal, 80% in Ethiopia and 90% in Nigeria were missing medical equipment. IUD removal data is not available for DRC.

**Fig 3:**
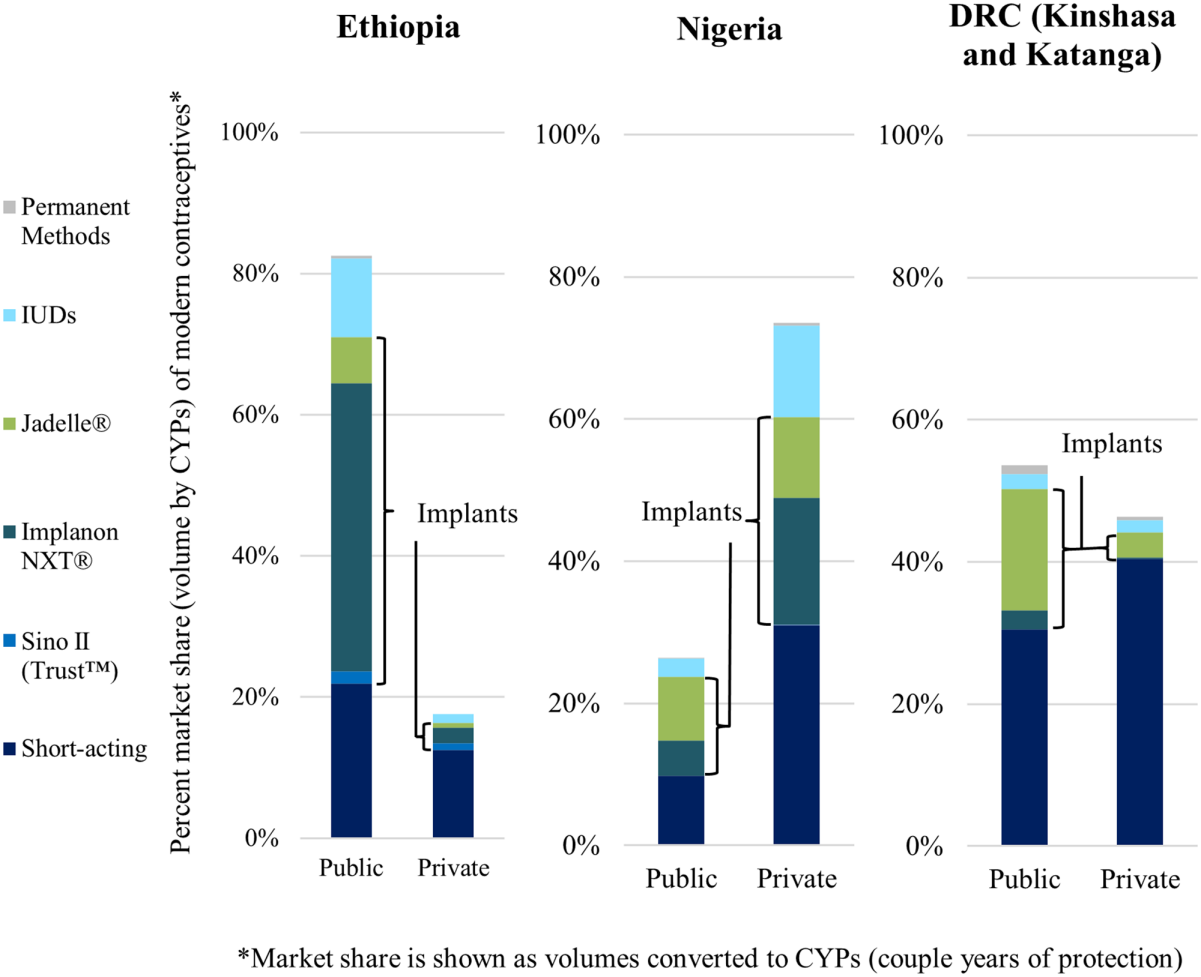
Market share of modern contraceptive methods across outlet types.

In Ethiopia, the public sector was responsible for more than 80% of total CYPs from contraceptives distributed, with LARCs making up the majority. Implants were 60% of public sector CYPs, but only 22% of private sector (Fig 3). Nigeria was the only country with more LARC CYPs in the private than public sector. Within the smaller public sector market, implants were 53% of CYPs and within the larger private sector they were 40%. While implant availability was low overall in DRC, most were provided in the public sector. Implants were 37% of DRC’s public sector CYPs and 8% of private sector. DRC was the only market where CYPs for short-acting methods were higher than for implants.

Implants made up a larger proportion of CYPs than IUDs across all countries and sectors; however, brand dominance differed between countries. Implanon NXT^®^ distributed by HEWs accounted for one-fifth of the total contraceptive market in Ethiopia and comprised 82% of the implant market in Ethiopia, compared to 13% for Jadelle^®^ and 5% for Sino II (Trust^™^). Implanon NXT^®^ and Jadelle^®^ had similar volumes in Nigeria (53% and 47% respectively). In DRC, Implanon NXT^®^ comprised only 12% of the implant market compared to 88% Jadelle^®^.

### LARC consumer pricing

**Fig 4:**
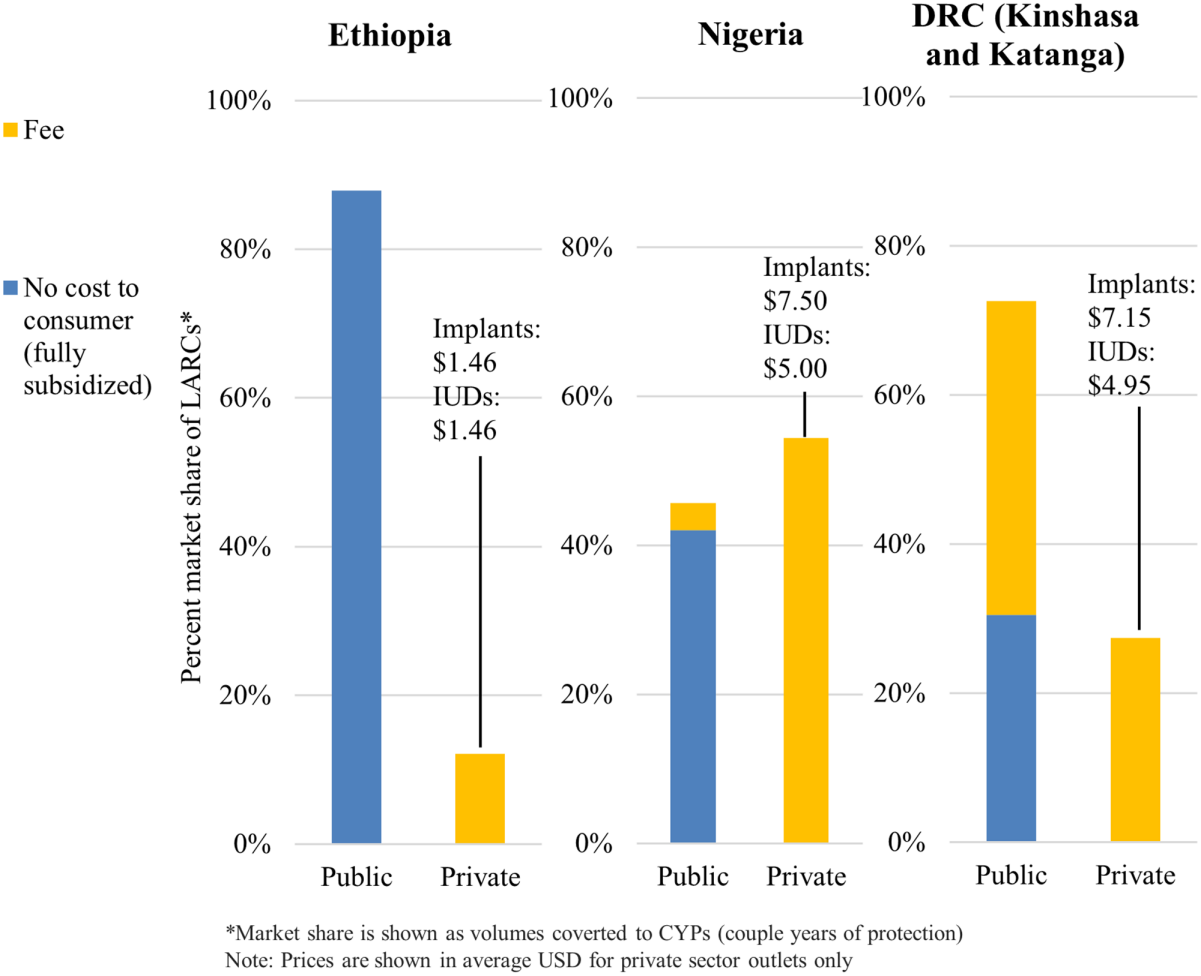
Proportion of LARCs distributed at no cost to the consumer or with a fee by sector and country.

In Ethiopia, LARC commodities and services were provided at no cost to the consumer in the public sector. In Nigeria, 92% of public sector outlets provided LARCs at no cost. In DRC, only 42% of public sector outlets provided LARCs at no cost, while 58% charged a fee for the contraceptive commodity, service, or both (Fig 4).

In Ethiopia, implants and IUDs sold in the private sector were comparable in price at $1.46. In Nigeria and DRC, implants were 50% more expensive than IUDs ($7.50 and $7.15 respectively compared to $5.00 and $4.95). In Ethiopia and Nigeria, LARCs tended to be more expensive in private clinics than in pharmacies and drug shops, likely due to an added service fee.

## Discussion

Limited availability of LARC methods restricts choice–barring women from contraceptive options that are easy to use consistently over time. [7] Ethiopia, where LARCs are readily available particularly in the public sector, has achieved mCPR growth. [21] In comparison, Nigeria and DRC, where LARCs are less available, have seen slower progress in mCPR. [21] PMA2020 monitoring demonstrates that this trend continues. [8] A policy and funding environment that models Ethiopia’s task shifting and widespread availability of implants has the potential for major impact on the contraceptive market and mCPR. Task shifting strategies already being piloted, as well as increased financial commitment, could bring Nigeria and DRC closer to reaching their FP2020 commitments and provide a roadmap for other countries in SSA.

When a range of LARCs is available within an outlet, implants are more likely to be distributed than IUDs. Other studies highlight why women might choose implants: they like the convenience and duration of contraceptive protection, and implants can be inserted without undergoing a pelvic exam. [7] Further, in Ethiopia where Jadelle^®^ and Implanon NXT^®^ have been widely available since 2005 and 2009, respectively, greater volumes of Implanon NXT^®^ were distributed in outlets that had both brands available. This could suggest a provider preference for implants with a prepackaged applicator over implants that require a trocar for insertion. In Nigeria and DRC, where introduction of Implanon NXT^®^ was more recent, the trend was weaker or not observed.

Substantial government investment is required to improve LARC availability and affordability. In 2013, the Jadelle^®^ Access Program and a similar initiative for Implanon NXT^®^ halved the price of implants for developing countries. [22] The Ethiopian government has taken advantage of these subsidies to purchase products in bulk and assure availability in public sector outlets. [16] Governments and donors can provide funding and sourcing support to increase the availability of implants at affordable prices in the public sector, but they must also have a sustainability plan. Such plans could include assuring private sector partners also have access to subsidized commodities, which would help generate demand for implants and expand the market.

In Ethiopia, brand-specific subsidies for Implanon NXT^®^, Jadelle^®^, and Sino II (Trust^™^) have lowered wholesale purchase prices. FPwatch data reflect implant availability in private clinics and pharmacies that is likely associated with this improved wholesale affordability. In all three countries, tiered pricing or additional point-of-provision subsidies in the private sector could make LARCs more affordable. Social marketing organizations could also help subsidize commodities and services and identify appropriate price points to improve affordability. [23]

Task shifting and increasing access to methods that require less equipment and training are promising strategies for increasing LARC uptake, especially in rural and underserved areas. Most outlets screened in Nigeria and DRC did not have LARC commodities or services available. Although the few outlets with LARC services tended to have credentialed providers, service readiness was curtailed by a lack of commodities and essential equipment. Task shifting provision of Implanon NXT^®^, which has fewer training and equipment requirements than other LARCs, has allowed HEWs to more than double the number of outlets providing LARCs in Ethiopia, reaching rural women who otherwise would not have access to contraceptives. [17] To manage removals, which require more equipment and a higher level of training, there is coordination with a linked health center that sends a credentialed provider to perform them in the community. [24] Expansion of implant and IUD provision by CHEWs in Nigeria [14] and pilots with medical and nursing students to administer Implanon NXT^®^ at the community level in DRC [15] could show similar results. In addition, stocking drug shops with LARCs, like DKT has done in Ethiopia, and training drug shop attendants to make referrals for LARCs, like PSI is currently doing in DRC, could leverage this existing infrastructure to fill commodity gaps.

### Strengths and limitations

To the best of our knowledge, no study has ever tracked contraceptive markets with such a large-scale, census-based approach as that used for FPwatch. We screened more than 25,000 outlets using a standardized survey format in Ethiopia, Nigeria, and DRC to understand contraceptive availability, market share, and price. We provide a comprehensive and current picture of where women are accessing LARCs and the potential of LARCs to help meet countries’ FP2020 commitments. The study had the following limitations: 1. Despite the large sample size, certain estimates by outlet type and strata resulted in small denominators; 2. Estimates were sometimes based on provider approximation rather than written records, which may have introduced recall bias to estimates for contraceptive volumes sold; 3. Due to the point-in-time nature of data collection, we were not able to confidently capture data for mobile outlets. This may have underestimated contraceptive estimates for areas of DRC and Nigeria; 4. While price indicators are standardized, differing economic realities make direct comparisons by country and even assumptions about affordability within countries difficult to precisely evaluate. 5. As a supply-side survey, examination of consumer demand and behavior was beyond the scope of the FPwatch study.

## Conclusion

FPwatch results highlight barriers to full contraceptive method access, including low availability, high prices, and lack of service readiness for LARCs. However, findings also suggest a market preference for implants and an affinity for the ease of insertion offered by Implanon NXT^®^, which saves time and effort on the part of providers and facilitates implementation of task shifting policies. Higher prices for LARCs limit access, but can be countered by subsidies that increase product affordability, as demonstrated in Ethiopia. Improving availability of LARCs, and particularly implants, is an important part of increasing voluntary contraceptive uptake. Shaping a regulatory environment that enables these strategies with consideration for the total contraceptive market is critical for reducing unmet contraceptive need in SSA and to reaching ambitious national and global FP2020 commitments.

## Supporting information

S1 Table FP2020 commitments and country contexts. (DOCX)

S2 Table Outlet categories and capabilities. (DOCX)

S3 Table Selected clusters by geopolitical zones in Nigeria and DRC (One-stage sampling). (DOCX)

S4 Table Selected clusters by geopolitical zones in Ethiopia (Two-stage sampling except for Addis Ababa). (DOCX)
