# Supplementary material for: Leveraging long acting reversible contraceptives to achieve FP2020 commitments in sub-Saharan Africa: The potential of implants
Source: PLoS One. 2018 Apr 9;13(4):e0195228. doi: 10.1371/journal.pone.0195228 (PMC5891008; doi:10.1371/journal.pone.0195228)
Supplement: S1 Table — (DOCX) [file pone.0195228.s001.docx]

**S1 Table. Supplemental Table 1. FP2020 commitments and country contexts.**

|  | **Ethiopia** | **Nigeria** | **DRC** |
| --- | --- | --- | --- |
| Contraceptive measures*  (MW= married women; UM= unmarried women) | -mCPR: 26·4%  -LARC mCPR: 9·9% MW, 11·5% UW  -Unmet need: 24·7% | -mCPR: 14·7%  -LARC mCPR: 1·5% MW, 1·4% UW  -Unmet need: 22·6 % | -mCPR: 10·0%  -LARC mCPR: 0·9% MW, 0·7% UW  -Unmet need: 40·8% |
| Current Method Mix | -LARCs: 15·2%  -Permanent: 0·4%  -Short-acting: 84·0%  -Other modern methods: 0·4% | -LARCs: 9·8%  -Permanent: 2·7%  -Short-acting: 82·2%  -Other modern methods: 5·4% | -LARCs: 7·6%  -Permanent: 6·3%  -Short-acting: 78·7%  -Other modern methods: 7·5% |
| FP2020 Commitments | -Increase contraceptive prevalence rate (CPR) to 55% by 2020  -Reduce total fertility rate (TFR) to 3 by 2020  -Reach an additional 6·2 million women and girls with family planning services  -Increase uptake of long-acting reversible methods  - Ensure commodities security  -Scale up delivery of services for the hardest to reach groups | -Increase CPR by 2% every year to achieve 36% by 2018  - Strengthen the in-country logistics system that ensures commodity availability at the facility level  - Train at least 3,700 community health workers (CHWs) to deliver the range of contraceptives, particularly long-acting and reversible methods and support task shifting so CHWs in rural areas can provide multiple methods· | -Increase the CPR from 6·5% in 2013 to 19% in 2020·  - Increase the number of users of modern methods from 700,000 in 2010 to 2·1 million in 2020  - Increase government funding dedicated to the purchase of contraceptives  - Reform laws which pose barriers to responsible parenthood and planned births  - Provide an increased range of at least 3 contraceptive methods in health facilities offering family planning: the extended range of contraceptive methods with at least 1 long-acting method |
| Financial Commitments | -Increase the family planning budget annually  To date:  -Reported 2015-16 budget for family planning (including human resources): US $27,551,187  -Conducted regional advocacy workshops to promote family planning as a major priority of health activities and budget allocation activities | -Provide an additional US $8.35 million to procuring reproductive health commodities from 2012-2016 (a 300% increase in funding)  -Work with the state and local governments to secure complementary budgets for family planning and reproductive health service delivery  -Meet health financing goals in the National Strategic Health Development Plan  -Meet or exceed the Abuja Declaration health financing commitments (spend 15% of government budget on health) | -Gradually increase the budget for purchase of contraceptives each year  To date:  -Established family planning and reproductive health as a government budget line item and allocated US $1 million to purchase of contraceptive in 2013 |

* The data for mCPR and unmet need were reported in the 2016 FP2020 Progress Report and were produced using the Track20 FPET model. Unmet need is shown for married women. The data for LARC mCPR and current method mix are from DHS reports in Ethiopia (2016), Nigeria (2013) and DRC (2013-14).
