# Supplementary material for: Leveraging long acting reversible contraceptives to achieve FP2020 commitments in sub-Saharan Africa: The potential of implants
Source: PLoS One. 2018 Apr 9;13(4):e0195228. doi: 10.1371/journal.pone.0195228 (PMC5891008; doi:10.1371/journal.pone.0195228)
Supplement: S2 Table — (DOCX) [file pone.0195228.s002.docx]

**S2 Table. Supplemental Table 2. Outlet categories and capabilities.**

| **Outlet**  **Category** | **Country** | | |
| --- | --- | --- | --- |
|  | **Ethiopia** | **Nigeria** | **DRC** |
| **Public Health Facilities** | - Public and not-for-profit facilities can provide implant and IUD services*. | - Public and not-for-profit facilities can provide implant and IUD services. | - Public and not-for-profit facilities can provide implant and IUD services. |
| **Community Health Workers** | - Health Posts are staffed by Health extension workers (HEWs) who can provide Implanon® implant insertion services**. | - Community health extension workers (CHEWs) have been piloting implant and IUD insertion and removal since 2015, however the program was not yet scaled up at the time of the survey. | - Relais communautaires are not equipped to provide LARC services. |
| **Private Clinics** | - Private hospitals and clinics can provide implant and IUD services. | - Private hospitals and clinics can provide implant and IUD services. | - Private hospitals and clinics can provide implant and IUD services. |
| **Pharmacies and Drug shops** | - Registered pharmacies***, drug shops and rural drug vendors (RDVs) can sell LARC commodities however neither can provide LARC services. | - Licensed pharmacies can sell LARC commodities however proprietary patent medicine vendors (PPMVs) cannot. Neither can provide LARC services. | - Licensed pharmacies and informal drug shops can sell LARC commodities however neither can provide LARC services. |

*Source: Ethiopia National Family Planning Guidelines (http://phe-ethiopia.org/resadmin/uploads/attachment-158- National_Family_planning%20guideline%20.pdf).

** IUD insertion and removal is now being piloted by level 4 HEWs but was not available in 2015.

***Registered pharmacies in Ethiopia are often attached to a private clinic that can provide services.

General retailers were excluded from this analysis since commodities above the level of condoms were not found.
