# Supplementary material for: Leveraging long acting reversible contraceptives to achieve FP2020 commitments in sub-Saharan Africa: The potential of implants
Source: PLoS One. 2018 Apr 9;13(4):e0195228. doi: 10.1371/journal.pone.0195228 (PMC5891008; doi:10.1371/journal.pone.0195228)
Supplement: S3 Table — (DOCX) [file pone.0195228.s003.docx]

**S3 Table. Supplemental Table 3. Selected Clusters by Geopolitical Zones in Nigeria and DRC (One-stage sampling).**

| **Country** | **Geopolitical Regions (Strata)** | **First Stage Selected Clusters** |
| --- | --- | --- |
| **Nigeria** |  | Localities |
|  | North Central | 58 |
|  | North East | 25 |
|  | North West | 29 |
|  | South East | 39 |
|  | South South | 30 |
|  | South West | 19 |
| **DRC** |  | Health Areas |
|  | Kinshasa | 34 |
|  | Katanga | 80 |
