# Supplementary material for: Leveraging long acting reversible contraceptives to achieve FP2020 commitments in sub-Saharan Africa: The potential of implants
Source: PLoS One. 2018 Apr 9;13(4):e0195228. doi: 10.1371/journal.pone.0195228 (PMC5891008; doi:10.1371/journal.pone.0195228)
Supplement: S4 Table — (DOCX) [file pone.0195228.s004.docx]

**S4 Table. Supplemental Table 4. Selected Clusters by Geopolitical Zones in Ethiopia (Two-stage sampling except for Addis Ababa).**

| **Country** | **Geopolitical Regions (Strata)** | **First Stage Selected Clusters** | **Second Stage Selected Clusters** |
| --- | --- | --- | --- |
| **Ethiopia** |  | **Woredas** | **Kebeles** |
|  | **Addis Ababa** | N/A^*^ | 27 |
|  | **Amhara** | 30 | 300 |
|  | **Oromia** | 30 | 300 |
|  | **SNNPR** | 33 | 330 |

^*^Addis Ababa does not have woredas so a single stage sample was taken at the level of the kebele using PPS
